# Doubled Haploids in Eggplant

**DOI:** 10.3390/biology10070685

**Published:** 2021-07-20

**Authors:** Ricardo Mir, Antonio Calabuig-Serna, Jose M. Seguí-Simarro

**Affiliations:** Cell Biology Group—COMAV Institute, Universitat Politècnica de València, 46011 Valencia, Spain; rimimo@upv.es (R.M.); ancaser3@upv.es (A.C.-S.)

**Keywords:** androgenesis, anther culture, aubergine, haploidy, microspore culture, Solanum melongena

## Abstract

**Simple Summary:**

This review compiles the most relevant advances made in the production of doubled haploid plants in eggplant, the application of doubled haploid lines in breeding programs, and the future perspectives for the development of alternative technologies for doubled haploid generation in this species.

**Abstract:**

Eggplant is a solanaceous crop cultivated worldwide for its edible fruit. Eggplant breeding programs are mainly aimed to the generation of F1 hybrids by crossing two highly homozygous, pure lines, which are traditionally obtained upon several self crossing generations, which is an expensive and time consuming process. Alternatively, fully homozygous, doubled haploid (DH) individuals can be induced from haploid cells of the germ line in a single generation. Several attempts have been made to develop protocols to produce eggplant DHs principally using anther culture and isolated microspore culture. Eggplant could be considered a moderately recalcitrant species in terms of ability for DH production. Anther culture stands nowadays as the most valuable technology to obtain eggplant DHs. However, the theoretical possibility of having plants regenerated from somatic tissues of the anther walls cannot be ruled out. For this reason, the use of isolated microspores is recommended when possible. This approach still has room for improvement, but it is largely genotype-dependent. In this review, we compile the most relevant advances made in DH production in eggplant, their application to breeding programs, and the future perspectives for the development of other, less genotype-dependent, DH technologies.

## 1. Introduction

Eggplant (*Solanum melongena* L.), also known as brinjal, aubergine, or Guinea squash, is a widely cultivated species for its edible fruits. In a broader sense, eggplant refers not only to *S. melongena*, but also to two other related African species, scarlet (*S. aethiopicum* L.), which is widely cultivated in Africa, and gboma (*S. macrocarpon* L.), a local species [1]. In this review, we will use “eggplant” to refer to *S. melongena.* Eggplant is one of the more than 3000 species belonging to the *Solanaceae* family. Several Asian regions such as India, different regions of China, or south-east Asian countries, have been proposed as the original domestication areas for this crop [2]. Among *Solanaceae*, eggplant is the third most cultivated species, after tomato and potato [3,4]. Indeed, eggplant cultivation currently covers 1.8 million hectares in the world, where nearly 55 million tons were produced in 2019 [4], with China and India being the main producers by far, followed by Egypt, Turkey, and Iran. Eggplant in the Mediterranean countries, as in Asia, is one of the top five most important vegetable crops [3]. Due to its economic importance, eggplant has been the focus of breeding programs. This has resulted in the generation of new varieties and hybrids, which entail a continuous rise in its production [5], which has doubled compared to twenty years ago with just a slight increase in cultivation area [4].

Hybrid seed production is based on the previous generation of pure, highly homozygous lines to be used as parentals. Pure lines have traditionally been obtained through several (6–10) self-crossing generations [6]. Eggplant is an autogamous species with large flowers that is easy to manipulate. Thus, it is technically feasible to obtain pure lines through conventional selfing and selection techniques. However, the time and costs required to generate new pure lines to test new F1 combinations are generally elevated [7,8]. Alternatively, the generation of homozygous, doubled haploid (DH) lines in a single generation can dramatically speed up this process, thereby becoming a convenient alternative to conventional approaches [9,10]. Currently, protocols for DH production have been described for almost 400 species [11] through the application of different in vivo and in vitro techniques such as pollination with irradiated pollen, interspecific and intraspecific crosses, crossing with natural or artificially produced haploid inducer lines, ovary or ovule in vitro culture, and isolated anther or microspore in vitro culture.

Anther and microspore culture are techniques used to induce embryogenesis in microspores, which are not naturally determined for this. It is therefore an experimental pathway that induces the development of microspore-derived embryos from haploid microspores under certain in vitro culture conditions. Since the first report of this phenomenon in *Datura innoxia* [12], protocols for many other species, including eggplant, have been reported [11]. Further genome duplication, artificially induced or not, results in the generation of fully homozygous DH individuals in a single generation [13]. The ability of microspores to develop as embryos, the so-called embryogenic response, is highly dependent on the genotype. Within the *Solanaceae* family, some tobacco lines show a very high embryogenic response, whereas tomato microspores are considered extremely recalcitrant to the induction of embryogenesis [8,13,14]. Despite this, although eggplant microspores are in general able to be induced to embryogenesis and generate haploid or DH embryos, they are still far from the efficiency of tobacco or other model species like *Brassica napus* in terms of embryogenic response.

In short, the generation of eggplant F1 hybrid varieties by crossing pure lines has favored the increment of eggplant productivity, as well as the improvement of other interesting agronomic traits. DH technology has the potential for accelerating the process of producing new parental pure lines in many different species, with eggplant considered a species moderately recalcitrant to the use of these techniques. Therefore, the optimization of DH protocols for this species is of general interest. Many different attempts have been made to develop new protocols or to improve the existing methods to produce DHs in this crop. To the best of our knowledge, all of them have explored microspore embryogenesis, either through anther culture or isolated microspore culture. A comparison of the main features of these two techniques is summarized in Table 1. In this review, we compile the main efforts made to generate eggplant DHs, the methods used to produce them and their use in breeding programs (summarized in Table 2). Finally, we discuss the future possibilities of DH technology in this species.

## 2. Anther Culture in Eggplant

Anther culture is the simplest technical method to induce microspore embryogenesis. The first report on the development of eggplant DHs by anther culture was published in 1973 [15]. This report described how anthers cultured in Nitsch’s medium supplemented with different concentrations of indole acetic acid (IAA) and kinetin developed DH plants from pollen-derived haploid calli and hypothesized that the change in the chromosome number was due to the spontaneous duplication of haploid cells during the early stages of callus proliferation. In 1978, the first haploid plantlets were obtained [16] and in 1979, the development of eggplant plantlets from microspore-derived calli through a combination of anther culture and microspore isolation was described [17]. It involved a pretreatment of the microspore-bearing anthers for four days and the posterior isolation of dividing microspores that, upon transference to Murashige and Skoog (MS) liquid medium supplemented with 2 mg/L 1,4-D and 1 mg/L kinetin, proliferated into calli from which plantlets were regenerated.

**Figure 1:**
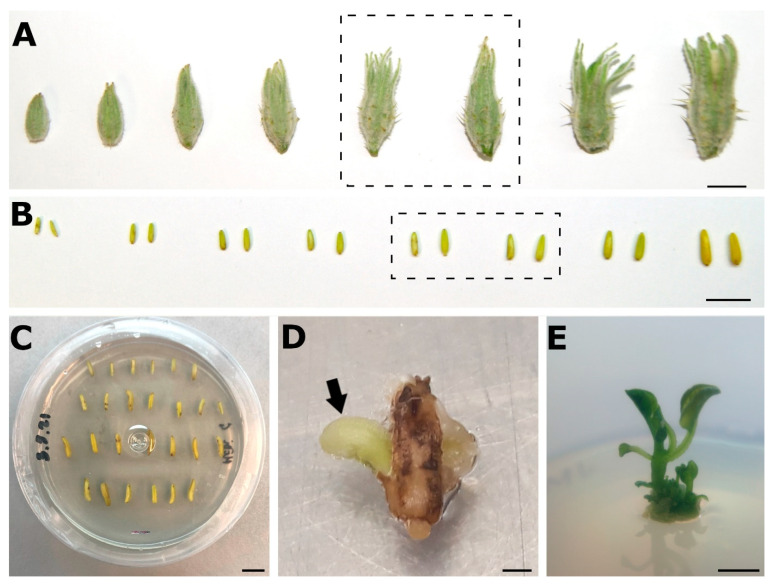
Anther culture in eggplant. (**A**). Range of developing eggplant floral buds. Developmental stages suitable for anther culture are boxed. (**B**). Range of eggplant anthers contained in the developing anthers shown in A. Anther sizes suitable for anther culture are boxed. (**C**). Anthers cultured in vitro on induction medium. (**D**). Microspore-derived embryo (arrow) emerging from a two month-old necrosing anther. (**E**). Eggplant plantlet produced in vitro from a germinated microspore-derived embryo. Bars: A–C, E: 1 cm; D: 1 mm.

The first reproducible protocol was described by Dumas de Vaulx and Chambonnet in 1982 [18]. Since then, it has been the most widely used technique (Figure 1). Briefly, it consists of the excision of flower buds at the right stage of development (Figure 1A) and, once in the laboratory, the extraction and surface-sterilization of anthers (Figure 1B), and their culture for 8 days at 35 °C in darkness in a semisolid induction medium supplemented with 0.01 mg/L kinetin and 0.01 mg/L 2,4-D (Figure 1C). Then, anthers are transferred to 25 °C with a 12/12 h day/night photoperiod for four more days, and then subcultured in a regeneration media supplemented with 0.1 mg/L kinetin and kept at 25 °C with a 12/12 h day/light photoperiod continuously. During the process, the anthers swell and necrose, while microspores proliferate within the pollen sac. Approximately two months later, microspore-derived embryos emerge from the anthers (Figure 1D). Then, they are removed from the anther and cultured until germination to produce a new microspore-derived in vitro plantlet (Figure 1E).

There are several factors affecting the embryogenic response of cultured anthers. The most important is the genotype. Different studies point to the notion that the ability of microspores to undergo embryogenesis is an inheritable and therefore genetically regulated trait, as will be discussed below in a separate section. The gene or genes controlling this trait, however, are still awaiting discovery. The second relevant parameter that determines a successful induction of microspore embryogenesis is the stage of the microspores used for in vitro culture, either through anther culture or through microspore culture. It is widely acknowledged that the best stage to induce embryogenesis revolves around the first pollen mitosis [13,33,36]. This means that, in general, mature, vacuolated microspores and young, just divided pollen grains are the stages where embryogenesis can be induced more efficiently. However, in the literature there are reports that suggest other stages slightly different from these. In some cases, the discrepancies may come from the difficulty of correlating anther sizes and microspore/pollen developmental stages. Sometimes, the difference in length between an anther with microspores at a given stage and an anther with microspores at the immediately later stage is as small as a tenth of a millimeter, which makes it very difficult to precisely pin it down. Besides, all microspores in an anther are not usually at the same stage. Instead, different stages coexist within the same anther [23]. Sometimes, the percentages of adjacent stages are very similar, which makes it difficult to draw clear and widely applicable conclusions. Eggplant is a particular example of a species where discrepancies in the suitable stage of microspores/pollen have also been published. In addition to the factors mentioned above, the thickness of the anther wall must also be taken into account when performing anther culture, as it influences the velocity at which media components reach microspores inside the locules [23]. It was proposed that, for anther culture, anthers should be excised when microspores are younger in order to allow time to let microspores progress until the suitable developmental stage while the growth factors of the culture medium diffuse through the thick anther walls and reach the anther locule. If anthers are excised when microspores are at the suitable stage, they will be too mature and therefore unresponsive by the time growth factors reach the anther locule.

The third main factor that influences the embryogenic response is the in vitro culture conditions, including the stress used to induce the developmental switch, and the composition of the culture medium. The Dumas de Vaulx and Chambonnet method is at present the basis for most of the anther culture protocols available in eggplant and, as to the stress used, very little has changed. A heat shock stress of 35 °C during several days is still the most used inductive treatment. However, different modifications have been proposed to adapt this method to improve its efficiency in specific eggplant varieties [37,38]. For example, the use of maltose was reported to be beneficial for embryogenesis induction, but also for plant regeneration, even in recalcitrant genotypes [20].

More recently, a triple combination of maltose, silver nitrate, and activated charcoal allowed for an increase in embryo yield of 3.9 times (production of up to 320 embryos and 200 plantlets/100 anthers) compared to the original medium [34]. The higher efficiency was associated with a positive synergistic effect of the three compounds in direct embryogenesis, embryo quality and other parameters related to the embryogenic response. In the same work, the first protocol for eggplant anther culture in liquid medium was developed, which resulted in up to 42 embryos per 100 anthers [34]. This medium was also based in the Dumas de Vaulx and Chambonnet method but supplemented with maltose and silver nitrate.

Further variations in Dumas de Vaulx protocol have shown that the treatment with other plant regulators during embryogenesis induction can also be efficient for the production of embryo-derived plantlets in certain cultivars. The replacement of kinetin by 1 mg/L zeatin riboside resulted in a percentage of responsive anthers of up to 27.8% [28]. It was also shown that there is a strong interaction between the genotype and the growth regulators used [29]. Other combinations of stress and induction medium have also been shown to be effective in anther culture. Embryo development was induced by preculturing anthers at 5–6 °C in MS medium supplemented with 1.0 mg/L 2,4-D and 1.0 mg/L kinetin [21]. The use of Gamborg (B5) salts as basal medium, supplemented with phenylacetic acid (PAA) was also found to be positive in terms of anther culture response [25].

Despite its advantages, anther culture still has some limitations. The most important is the possibility, at least theoretically, of inducing the occurrence of somatic embryos developed from anther tissues other than microspores. In parallel to the development of microspore-derived embryos, calli may emerge from the anthers upon exposure of the anther to the in vitro culture conditions [39]. These calli may have either microspore or somatic origin. Thus, although the first could be used to regenerate haploid or DH plants, it would be necessary to analyze their origin with molecular markers in order to identify somatic calli and discard them. Additionally, somatic calli consume resources and space, which may affect the growth of true microspore-derived embryos. The region of the wound produced when anthers are excised from the filament is especially prone to proliferation as undifferentiated callus masses.

An additional problem is the lack of control of the substances secreted by the anther wall layers surrounding the pollen sac. Some of them may have a positive effect on the induction and regeneration of androgenic embryos, but many others, principally coming from the degradation of these tissues, may have detrimental and undesired effects [8]. In many cases, these problems may have a relative effect on the final yield of DHs, and there may not be a need for solving them. However, in other cases these limitations make anther culture inefficient. In these cases, isolated microspore culture may constitute a suitable alternative.

### In Vitro Culture of Isolated Eggplant Microspores

In isolated microspore cultures, microspores/young pollen are isolated from the donor anthers. They are the only cell types present in the culture and, therefore, the only possible origin for the regenerated plants. Other advantages of microspore cultures include an increase in the efficiency of embryo production, since embryos are not constrained by the reduced space of the anther locule, and a better control of medium conditions, since no other tissues are present and cannot modify the medium composition by the secretion of beneficial or harmful compounds. Freely suspended microspores are also amenable for transformation by biolistic techniques, or combined biolistic and *Agrobacterium*-mediated transformation, by using plasmids, short peptide nanocarriers and cell penetrating peptides [40,41,42].

**Figure 2:**
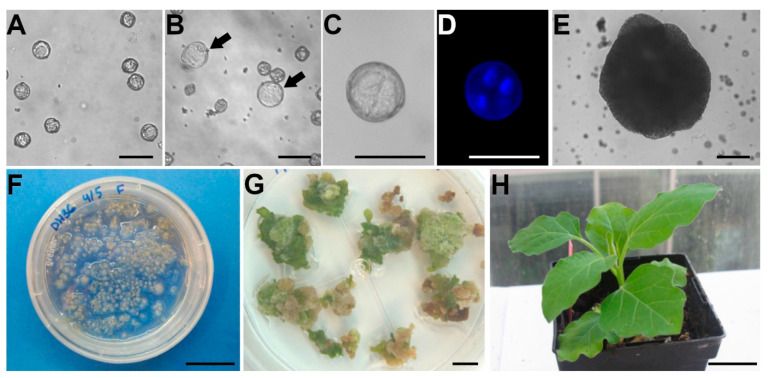
Isolated microspore culture in eggplant. (**A**). Microspores freshly isolated from eggplant anthers and cultured in vitro in liquid medium. (**B**). Cultured microspores after the induction treatment. Most microspores remain arrested, with no evident development, whereas others enlarge (arrows) as a consequence of embryogenesis induction. (**C**,**D**). Multicellular microspore-derived structure as seen under phase contrast (**C**) and DAPI staining for visualization of the nuclei (**D**). (**E**). Callus-like structure formed from a microspore-derived embryo in vitro culture. (**F**). Culture dish with microspore-derived calli. (**G**). Regenerating calli where multiple green, organogenic nodules are seen on their surface. (**H**). Acclimatized eggplant DH plant obtained through microspore culture. Bars: A–E: 50 µm; F, G: 1 cm; H: 5 cm.

In the first attempt to establish an isolated microspore culture protocol in eggplant, isolated late uninucleated microspores and young pollen grains belonging to three different F1 cultivars were subjected to a combination of both starvation and heat stress, which resulted in the generation of microspore-derived structures with an efficiency higher than by anther culture-based protocols [19]. This protocol, however, produced no embryos but calli with different levels of ploidy and a limited ability for shoot regeneration from them. This initial protocol has served as the basis for the different modifications made subsequently. Briefly, isolated microspores (Figure 2A) are incubated at 35 °C during three days suspended in distilled water under dark conditions. The combination of heat stress and full nutrient starvation promotes the developmental switch in a percentage of microspores higher than with anther culture. Such a switch is revealed by an increase in size of induced microspores (Figure 2B), whereas the non-induced cells stop their growth and/or die. After the stress treatment, microspores are transferred to an NLN-based medium supplemented with 6-benzylaminopurine (BA) and naphthaleneacetic acid (NAA) and cultured continuously at 25 °C in darkness. After 7–10 days, depending on the genotype and/or the growth conditions of donor plants, some microspores will show organized embryogenic cell divisions (Figure 2C,D) that end up with the formation of globular-like embryos. However, to our knowledge, it has not been possible up to now to promote the development of eggplant microspore-derived embryos beyond this stage up to the mature, cotyledonary stage in a reproducible manner. Microspore-derived structures keep their embryo identity up to the globular stage [24] and then they transform into proliferative but undifferentiated callus-like structures (Figure 2E,F). This situation is opposed to that of other species such as, for example, *Brassica napus* or tobacco, which develop true embryos. Regardless, these calli can be subcultured in regeneration medium to induce shoot and root organogenesis (Figure 2G) and, after acclimatization of the in vitro plants produced, fully functional DHs are obtained (Figure 2H).

Despite the economic importance of eggplant, modifications of the different parts of this protocol to make it more efficient were not proposed until more than fifteen years later. The first aspect that influences the embryogenic response of microspores is the conditions under which the donor plants have been cultured, which influences further viability of microspores to a large extent. Indeed, the viability of eggplant microspores was found to be affected by the light intensity applied to donor plants, but not by slight (circa 6 °C) temperature changes [35]. Interestingly, the viability of eggplant microspores was not dependent on light intensity at the moment of excision of the floral buds, but it was influenced by the amount of light that donor plants were exposed to during the two days prior to excision [35]. Moreover, although microelements such as boron has been shown to increase pollen quality in pepper [43], the foliar application of boron at low doses produced no effects in the viability of eggplant microspores, and notably reduced it at high doses [35].

Once microspores are isolated and prior to culture initiation, microspores must be suspended in the liquid medium at a defined density in order to optimize the efficiency. A microspore density between 1 × 10^5^ and 5 × 10^5^ microspores/mL was initially proposed [19]. Recently, a more detailed assay was performed to narrow down this range of cell densities [35]. The highest number of microspore-derived calli was produced when microspores were cultured at densities between 2 × 10^5^ and 3 × 10^5^. Lower densities (i.e., 5 × 10^4^) produced no calli at all. In turn, higher densities (i.e., 2 × 10^6^) resulted systematically in the growth of bacterial colonies, possibly endophytes released from anther tissues during the process of microspore isolation.

Efforts have also been devoted to the optimization of in vitro culture conditions. In different eggplant genotypes, positive effects on the embryogenic response have been observed with different modifications of the initial culture medium, including the addition, alone or in combination, of growth regulators such as abscisic acid and epibrassinolide, and of biopolymers such as polyethylene glycol, arabinogalactans and arabinogalactan proteins [26]. Additionally, a reduction in NAA and BA was found to be beneficial to increase the rate of embryogenesis induction [26]. However, the main bottleneck of microspore embryogenesis through microspore culture in eggplant is still the difficulty of microspore-derived embryos in undergoing a true transition from globular to bipolar, differentiated embryos. Although the exact reason for the development of callus-like structures is still unknown, it seems likely due to a suboptimal medium composition where some components, probably growth regulators, are not properly balanced. In this sense, a reduction in growth regulators in the culture medium has been shown to increase the occurrence of structures anatomically closer to true embryos, rather than to callus-like structures (manuscript in preparation). Surely, future research should focus on this stage. Finally, plant regeneration through shoot and root organogenesis has also been improved using different relative amounts of IAA and zeatin in the regeneration medium, thereby increasing the frequency of organogenesis [24,27].

## 3. The Critical Role of the Genotype in the Embryogenic Response of Eggplant Microspores

Aside from the growth conditions of donor plants and the particular in vitro culture conditions used, the most determinant factor for an efficient production of androgenic DHs, either by anther culture or by isolated microspore culture, is the genotype of donor plants. In all the species studied, there are varieties or genotypes that are responsive to embryogenesis whereas others are very recalcitrant. In the case of eggplant, a study of 12 different accessions of common eggplant and related materials from the primary genepool (the eggplant complex) and the secondary genepool showed that only common eggplant (*Solanum melongena*) materials responded to microspore embryogenesis [39]. Wild relatives and even their crosses with common eggplant materials showed no response. This is not surprising, since microspore embryogenesis does not occur in nature, and wild species have not been selected during their evolution to become more efficient for this trait. Instead, it is possible that some of the responding genotypes, in particular the F_1_ hybrids, were subjected to anther culture during the breeding programs they come from. Indeed, the two best performing genotypes of the study (Bandera and Ecavi) are commercial F_1_ hybrids [39]. This was confirmed in a later study where microspores of these accessions were isolated and cultured in vitro. The three responding genotypes were Bandera, Ecavi, and Cristal, the three commercial F_1_ hybrids tested [24].

As for any other genetic trait, the embryogenic competence of eggplant microspores segregates in the offspring of F_1_ hybrids. Indeed, an eggplant DH population was developed from the F_1_ hybrid Bandera [30]. It was found that, in addition to segregation of several morphological and reproductive traits, the androgenic response also showed variation among DH lines, from null to very high. One of these lines (DH36) was very similar to Bandera in anatomy and reproductive competence but produced up to four times more microspore-derived calli than Bandera in a stable manner. DH36 has then been used for other studies aimed at optimizing the protocol of microspore culture. It was found that its response to changes in the in vitro conditions differed from other F_1_ hybrids, possibly due to the different steps of in vitro selection undergone by this line. For example, it was found that when the concentration of basal salts and sucrose was increased and the concentration of growth regulators was reduced, the number of microspore-derived calli produced by DH36 decreased, whereas it was remarkably increased in the two different F_1_ hybrids [35]. These studies demonstrated the genetic basis of the embryogenic competence of eggplant microspores and illustrated how this trait can be improved. However, only few data are available about the cellular, molecular, or physiological basis for embryogenic competence in eggplant.

One of the reasons for the lower embryogenic response of eggplant, when compared to other species such as *B. napus*, may relate to intracellular Ca^2+^ levels. A peak of Ca^2+^ accumulation was observed in vacuolated microspores and young pollen (the stages inducible to embryogenesis) of the highly embryogenic *B. napus* DH4079 line, whereas in equivalent stages of the eggplant DH36 line, Ca^2+^ levels were much lower [31]. Later on it was found that a callose-rich subintinal layer was formed in embryogenic microspores of both *B. napus* and eggplant lines, but it was thicker and richer in callose in microspores of the highly embryogenic *B. napus* line and thinner and less callose-rich in eggplant microspores [32]. Microspores of the high response *B. napus* line with thicker subintinal layers exhibited a higher protection against osmotic stress and increased viability when cultured in vitro in liquid medium [32]. Thus, it seemed that the subintinal layer confers a protection against osmotic stress at least, which increases the viability of in vitro cultured microspores and therefore their chance to become embryogenic. A low response *B. napus* line (DH12075) was also analyzed in parallel with the high response *B. napus* DH4079 line and the DH36 eggplant line. Interestingly, the results of the low response *B. napus* line were remarkably similar to those of the eggplant line, both showing lower Ca^2+^ levels and thinner subintinal layers with less callose and lower embryogenic competence than the high response *B. napus* line [32].

These findings relate the presence of the callose-rich subintinal layer with the embryogenic competence, being greater in high-response genotypes. Callose deposition in the subintinal layer was found to be Ca^2+^-dependent [32], which established a link between high Ca^2+^ levels in the microspore/pollen stages inducible to embryogenesis, the formation of a callose-rich, protective subintinal layer, and a high embryogenic response. In the case of eggplant, lower Ca^2+^ levels in the inducible stages would account for the formation of a thinner subintinal layer, where callose is deposited in lower amounts. Such a layer in eggplant microspores would be less protective against osmotic stress, and this would explain the dramatic decrease in viability observed in cultured microspores after the inductive treatment (our unpublished observations) and their eventual lower embryogenic competence. According to this, it could be speculated that, as in *B. napus*, the differences in embryogenic response among eggplant genotypes could be related to similarly different levels of intracellular Ca^2+^ and therefore a different ability to form a protective subintinal layer.

## 4. Genome Doubling of Haploid Individuals

Microspore-derived haploid embryos can produce weak and sterile plants. To prevent this, their chromosome set, whether maternal (gynogenesis) or paternal (androgenesis), must undergo genome duplication in order to become true DHs [44,45]. Mainly, cellular processes such as nuclear fusion are the cause of spontaneous genome doubling [45,46], and their occurrence is highly dependent on plant species. For instance, species such as *Hordeum vulgare* [47], *Sorghum bicolor* [48], *Brassica oleraceae* var *italica* [49] and *Solanum tuberosum* [50] show a direct, spontaneous genome doubling rate higher than 90%, whereas species like *Triticum aestivum* [51], *Oryza sativa* [52,53], *Cucumis sativus* [54], *Cucumis melo* [53], *Allium cepa* [55], or *Beta vulgaris* [56,57] are recalcitrant to genome doubling. Nevertheless, protocols to induce indirect genome doubling with the aid of drugs are needed for both types of species in order to efficiently exploit the potential of DH technology.

Indirect genome doubling protocols are mainly based on the application of antimitotic compounds that inhibit microtubule polymerization, resulting in defective microtubule-based structures (the mitotic spindle and the phragmoplast) and eventually in abnormal chromosome segregation when applied at high doses and in nuclear fusion when applied at low doses [45]. Colchicine is a naturally occurring, antimitotic compound traditionally used to induce polyploidy in diploid species, and to induce doubled haploidy in haploid individuals. In fact, it is the preferred drug for genome doubling in haploids. However, its toxic effects on animal (including human) cells and for the environment has boosted the search for other antimitotic compounds, such as the dinitroaniline-based herbicides oryzalin, trifluralin and amiprophos-methyl. Specific protocols with different drugs, doses, and application modes under in vitro and in vivo conditions have been recently reviewed [44,58]. Specifically, eggplant shows a moderate ability to undergo genome doubling without using any drug. In some backgrounds a percentage close to 45% was reported [34], whereas in others it reached 60% [24]. Genome doubling in eggplant seems also to be influenced by the in vitro culture time of haploid seedlings, so the longer they remain in vitro, the higher the doubling rates [39].

The protocols for induction of genome doubling when haploid plants are still in vitro typically include the use of 0.5–1% aqueous solutions of colchicine. For example, the best performing treatments for eight different eggplant backgrounds were reported to be 0.5% colchicine for 2 h and 1% colchicine for 1 h [22]. For acclimated, ex vitro haploid plants, the application of 0.5% colchicine dissolved in lanolin paste for two days has been reported successful to induce doubling in axillary buds [59]. Thick lanolin paste is used to avoid liquid colchicine evaporation and draining away from the bud. With this method it was possible to increase the production of DH plants by an additional 25% with respect to the rate of direct genome doubling [24].

Moreover, in our experience, the rate of direct genome doubling without using antimitotic drugs varies enormously among eggplant materials. When using new, untested materials, the most reasonable starting point would be to evaluate what the direct genome doubling rate is, and only when it does not meet the particular needs of the experiments, try different combinations of colchicine concentrations and times. If colchicine does not produce the necessary frequency of genome doubling in DH plants, or if special regulations against the use of colchicine operate, other antimitotics should be tried.

## 5. Use of DHs for Eggplant Breeding Programs

Doubled haploid (DH) technology for plant breeding has been around since the 1970s, and this technology was used to obtain DH lines initially in *B. napus* [60] and barley [61]. From these pioneer findings, androgenic DHs have been used to produce commercial varieties in many other species such as wheat, other *Brassica* species, rice, melon, asparagus, as well as *Solanaceae* including pepper, tobacco, or eggplant, which has resulted in the generation of more than 300 new varieties [62,63]. Eggplant breeding programs are mostly focused on the release of hybrid varieties with improved traits [59]. Eggplant F1 hybrids, resulting from the crossing of two parental homozygous plants, typically perform better than parental lines for different agronomic traits [5,64]. A great amount of effort has been devoted to evaluating the performance of eggplant F1 hybrids for different characteristics, using wild or local homozygous landraces as parental lines [5,65,66,67]. Among others, the valuable agronomic objective of eggplant breeding programs are yield, fruit color, seed/pulp ratio, flesh consistency and browning, nutritional characteristics, and resistance to diseases [59].

Breeding for disease resistance is important since commercial eggplant cultivars are known to have limited resistance to disease [68]. In this context, anther culture, the DH technology most developed and widely applicable in eggplant, has been routinely used by private seed companies since the end of the 1980s, soon after the discovery of the method of Dumas de Vaulx and Chambonnet, to produce pure lines as part of their eggplant breeding programs [59,68]. It is therefore likely that many of the current varieties have benefited from this technique. Recently driven by the challenge of climate change, there has been an increasing interest in eggplant research, principally focused on the use of wild eggplant relatives to develop new varieties with improved resistance to bacterial wilt, as well as drought and heat, which will surely contribute to the generation of new and improved cultivars.

Anther culture has also been used to reduce the ploidy of tetraploids, thereby producing dihaploid progenies as, for example, in tetraploid interspecific hybrids *S. melongena* × *S. integrifolium* and *S. melongena* cv. Dourga × *S. aethiopicum* [69,70,71,72]. The dihaploid lines were then used to introgress *Fusarium oxysporum* resistance genes and their nutraceutical and health-promoting compounds were characterized, proving successful as an approach to obtain new eggplant genotypes with useful traits derived from related species.

Other than the generation of eggplant hybrid lines, DH technology has contributed to both the mapping of interesting agronomical traits and the generation of genetic variability. For example, DH populations have been used to detect quantitative trait loci (QTLs) related with resistance to *Ralstonia pseudosolanacearum* [73], and yield-related loci [74]. Genetic variation among eggplant DHs lines is produced using both inbred cultivars and heterozygous lines as donor plants [59]. Specifically, the phenotypic segregation of leaf, flower, and fruit traits, including fruit set, seed setting, and germination rate, was studied in a DH population obtained from a commercial F_1_ hybrid [30]. Moderate morphological variability was observed in this study. The presence of prickles in the fruit calix was found to be one of the most variable phenotypes, as individuals from the DH population showed significantly fewer prickles than the F_1_ donor line. Additionally, flower color, the number of flowers per inflorescence, and primary and secondary fruit colors presented moderate to high variability. The DH population also showed a slightly reduced reproductive ability when selfed compared with the F_1_ [30]. On the other hand, a study of genes from *Solanum aethiopicum* introgressed in *S. melongena* revealed that anther cultures generate DH populations with massive segregation distortion, which limits the usefulness of the method to preserve all the genetic variation of the parental lines [75].

In summary, DH technology and principally anther culture still has some practical limitations when applied to eggplant breeding. However, DH technology has shown substantial potential to assist breeders, not only for the generation of hybrid varieties, but also to gain genetic variability and to identify QTLs associated with agronomically important traits. Therefore, it is expected to be used in the future as part of current breeding strategies to deal with increasing food demand, the emergence of new pests, as well as biotic and abiotic stresses caused by global warming.

## 6. Limitations of DH Technology and Future Perspectives

To the best of our knowledge, the only DH technologies that have consistently yielded positive results in eggplant have been microspore culture and, principally, anther culture. Eggplant DH populations produced by these methods must generate sufficient genetic variability to be useful to produce pure lines for hybrid seed production, and the possible occurrence of significant segregation distortion should be evaluated in each DH population. These two techniques, based on in vitro androgenesis, also present the limitations typical of any other in vitro culture process. The most important, as mentioned above, is the genotype. In addition, the low efficiency of embryo induction and the need for checking the origin of all the plants regenerated from anther cultures, and the current inability to obtain mature, germinating microspore-derived embryos from microspore cultures, have restricted the practical large-scale implementation of these methods for hybrid seed production. Finally, the limited knowledge of the cellular, molecular, and genetic mechanisms controlling recalcitrance in different genotypes is also a relevant obstacle to increase the efficiency of these processes. These techniques still have room for improvement, principally microspore culture and the undesirable transformation of microspore-derived embryos into calli. However, microspore embryogenesis will always depend on the genotype of donor plants to a large extent.

As an alternative to in vitro-based approaches, the possibility of generating haploid inducer lines seems more and more feasible. In the last decade, the manipulation of certain genes has allowed for the production of haploid inducer lines in different species. In maize, mutations in genes like *NOT LIKE DAD (NLD)/MATRILINEAL (MATL)/patatin-like phospholipase-A1 (PLA1)* or *DOMAIN OF UNKNOWN FUNCTION 679 (DMP)* have consistently generated lines that, when crossed with elite materials, produce haploids with the genome of the elite line [76,77]. Unfortunately, *NLD/MATL/ZmPLA1* does not have homologs in dicots, but the dicot *Arabidopsis thaliana* has multiple DMP-like homologs, and one of the double mutants generated (*atdmp8–/–atdmp9–/–)* was shown to induce haploids when crossed [78]. It seems reasonable that other dicots such as eggplant may also have *DMP*-like homologs.

However, the most promising gene to mutate in order to generate haploid inducer lines in dicots such as eggplant is the gene of the centromeric histone H3 (*CENH3*). This approach was first described in *Arabidopsis thaliana* [79]. It consists of editing the centromere-targeting domain of CENH3 and the crossing of these mutants with wild type individuals. Then, during the early stages of embryogenesis, the mutant genome with inactivated centromeres is eliminated and an embryo bearing only the chromosomes from the wild-type parental is developed [80]. The *cenh3* mutants were first produced by genetic transformation, but they are currently also produced through gene editing and also by EMS-driven mutagenesis [81]. Haploid-producing *cenh3* mutants have already been reported for different crops, including maize and wheat [82,83,84], and the method has also been tested in red cabbage [85], barley, and sugar beet [86]. There are also patents and inventions that claim to have developed similar systems in other agronomically interesting crops by *CENH3* manipulation, but also by alteration of other components of the kinetochore complex (reviewed in [80]).

As seen, intensive research is being done in this field as this system could be potentially useful to obtain haploids and then DHs in species described as recalcitrant for anther or microspore cultures [87]. In this sense, eggplant could be a suitable candidate, as its genome has already been recently sequenced and assembled [88,89], and there are already protocols available for genetic transformation (reviewed in [90,91]) and gene editing [92]. This alternative could overcome the limitations imposed by the genotype for in vitro-based methods, the only approaches explored so far, and could therefore be useful for a broader range of eggplant materials.

Despite the possibilities of these promising technologies, the final implementation of these techniques to assist in eggplant breeding programs will eventually be, as in many other cases in applied research, dependent on funding. Each seed company should determine to what extent the economic return of the use of these techniques in their eggplant programs justifies the initial investments that must be made to implement them. In addition, legal considerations must also be taken into account, at least in the case of gene editing. Although it is authorized in some countries worldwide, in others, such as those of the European Union, they are still banned. It appears paradoxical in a case like this, where the edited mutant is only used as an inducer, and the final product does not even contain a single trace of the edited genes.

## 7. Conclusions

Anther culture is the most widely used and easy method to produce DH plants in eggplant. Isolated microspore culture, although more technically challenging, has additional advantages that make it worth implementing. However, problems such as the transformation of embryos into calli and the identification of an efficient and universal method for genome doubling are still to be solved. In recent decades, DH technology (anther culture principally) has been used in different eggplant breeding programs. Eggplant could be a suitable candidate for the future development of haploid inducer lines through CRISPR/Cas9 gene editing.

## Figures and Tables

**Table 1:** Comparison of anther and isolated microspore in vitro culture in eggplant.

	Anther Culture	Microspore Culture
Technical complexity	Lower	Higher
Applicability	Higher	Lower
Control of medium composition	Lower	Higher
Efficiency	Lower	Higher
Speed	Slower	Faster
Need for checking haploid origin	Yes	No
Microspore transformation	Not possible	Possible
Output	Microspore-derived embryos	Microspore-derived calli

**Table 2:** Chronological advances in the protocol for eggplant DH production through anther and isolated microspore culture. a: anther culture, m: microspore culture.

Technique	Year	Advance
a	1975 [15]	First report on eggplant anther culture.
a	1978 [16]	First eggplant DH plantlets.
a, m	1979 [17]	Combination of eggplant anther culture and microspore isolation.
a	1982 [18]	First reproducible eggplant anther culture protocol.
m	1996 [19]	First report of eggplant microspore culture.
a	2006 [20]	Effect of maltose in the embryogenic response.
a	2008 [21]	Effect of cold stress preculture in the embryogenic response.
m	2011 [22]	Colchicine-based genome doubling protocol.
a	2012 [23]	Effect of stage for anther excision and heterostyly.
m	2012 [24]	Improved protocol for microspore-derived callus production by starvation and heat stress.
a	2013 [25]	Study of the effect of PAA and Gamborg (B5) salts.
m	2014 [26]	Increased efficiency of microspore culture through modifications of the culture medium.
m	2015 [27]	Optimization of plant regeneration from microspore-derived calli.
a	2017 [28]	Protocol improvement by replacement of kinetin by zeatin riboside.
a	2017 [29]	Interaction between genotype and growth regulators.
m	2017 [30]	Development of a DH line with high embryogenic response.
m	2017 [31]	Role of calcium in microspore embryogenesis.
m	2019 [32]	Role of the cell wall in the embryogenic response of different species, including eggplant.
m	2020 [33]	Procedure for the identification of the microspore/pollen responsive stages.
a	2020 [34]	Effect of maltose, silver nitrate and activated charcoal in the embryogenic response. Establishment of a protocol for anther culture in liquid medium.
m	2020 [35]	Effect of light intensity over donor plants and in vitro microspore density in the embryogenic response.

## Data Availability

Data sharing is not applicable to this article.
